# Analysis on the situation of subjective well-being and its influencing factors in patients with ankylosing spondylitis

**DOI:** 10.1186/s12955-016-0522-7

**Published:** 2016-08-22

**Authors:** Mengmeng Wang, Sheng Wang, Xu Zhang, Qing Xia, Guoqi Cai, Xiao Yang, Xiaona Li, Li Wang, Lihong Xin, Shengqian Xu, Jianhua Xu, Zongwen Shuai, Faming Pan

**Affiliations:** 1 Department of Epidemiology and Biostatistics, School of Public Health, Anhui Medical University, 81 Meishan Road, Hefei, Anhui 230032 China; 2 Department of Rheumatism and Immunity, the First Affiliated Hospital of Anhui Medical University, Hefei, Anhui 230022 China

**Keywords:** Ankylosing spondylitis, Influencing factors, Subjective well-being

## Abstract

**Background:**

To examine the subjective well-being (SWB) in patients with ankylosing spondylitis (AS) compared with the healthy controls, and to explore the associations between SWB and demographic characteristics, disease-specific variables in AS patients.

**Methods:**

SWB was assessed with General Well-Being Schedule (GWBS) in 200 AS patients and 210 healthy controls. Comparisons among subgroups were performed to investigate how certain aspects operate as favorable or adverse factors in influencing SWB in the patients with AS.

**Results:**

Both men and women with AS reported significantly impaired SWB on all scales of the GWBS except for the Control (O) scale. The results revealed that better sleep, lower disease activity and more family care predicted higher SWB. In AS patients, positive attitude towards therapy prospect was significantly associated with higher SWB. Therapy prospect refers to the hope of patients about the disease treatment.

**Conclusions:**

Compared with general population, SWB might be affected by the onset of AS. There are significant associations between SWB and sleep quality, BASDAI, APGAR, therapy prospect.

## Background

Ankylosing spondylitis (AS) is a chronic inflammatory disease that affects the sacroiliac joints and spine of young adults, especially men (sex ratio 2:1) [1] and its prevalence is most commonly reported to be 0.1 to 1.4 % depending on the population [2]. People who are affected generally present at around 26 years of age, typically have inflammatory back pain and structural damage, resulting in joint stiffness and a gradual loss of spinal mobility [2–4]. Male patients have more functional limitations and lower bone mineral density than female patients [5]. Because of the early onset, related work disability, absence from paid work and socioeconomic burden to individuals with AS are substantial especially men [6–8]. As these is no radical cure for AS patients at present, an important goal in treatment is to control the spinal inflammation and the resultant pain and stiffness [9].

Subjective well-being (SWB), a popular concept in positive psychology, has gained increasing attention in medical science [10]. Higher subjective well-being helps people to be more energetic, which is a vital component in recovery and treatment [11]. With increasing progress in improving functional capacity and survival in AS patients, it is becoming increasingly clear that, for many AS sufferers, improving the quality of life is equally as important as the survival benefit provided by pharmacological treatment. SWB refers to subjective and multidimensional evaluation of daily life [12, 13], which can be measured by the General Well-Being Schedule (GWBS). GWBS is a generic instrument, which covers the most central dimensions of subjective health and applies to diseased populations and healthy people. As we all know, there are many schedules regarding quality of life such as EuroQol (EQ-5D) or Short Form-36 (SF-36). However, the above schedules include physical components and mental components, which are used to reflect physical health and psychological health respectively [14, 15]. Compared with EQ-5D or SF-36, the General Well-Being Schedule (GWBS) is a brief indicator of subjective feelings of psychological well-being and reflects mental health totally [16]. Furthermore, there are many studies about SF-36 evaluation in AS patients, but few about GWBS. Our team has verified the health-related quality of life of ankylosing spondylitis patients assessed by SF-36 [17]. Thus, we choose the GWBS in this current study.

The previous studies on subjective quality of life mainly aimed at healthy people, empirical knowledge on the subjective health of AS patients is relatively scarce. The aim of this study is to assess the SWB in patients with AS compared with the healthy controls, and to investigate the relationship of various domains of SWB with demographic characteristics, disease-specific variables in AS.

## Methods

### Participants

Patients with AS participating in this study were recruited from the Department of Rheumatology, the First Affiliated Hospital of Anhui Medical University between 2013 and 2014. There are 200 AS patients fulfilling the New York classification criteria [18]. A total of 210 sex and age matched healthy controls were collected from Physical Examination Center, the First Affiliated Hospital of Anhui Medical University.

### Data collection

The study group included AS patients and healthy controls, they received a questionnaire including demographic variables, APGAR, some disease-specific instruments and a generic instrument (GWBS). SWB was assessed by the generic instrument (GWBS) in patients with AS and healthy controls. In healthy controls, it is unnecessary to complete the part of disease-specific instruments. All questionnaires were completed with the help of specialized training investigators. We obtained informed consent from all the patients and healthy controls and the study was approved by the ethical committee for medical research.

### Adaptation, Partner-ship, Growth, Affection and Resolve (APGAR)

APGAR meaning satisfaction with family function was evaluated by the Family APGAR Index, which was compiled by Smilkstein, including 5 items: adaptation, partner-ship, growth, affection and resolve [19]. These five items corresponded to the following questions, I am satisfied that I can turn to my family for help when something is troubling me; I am satisfied with the way my family talks over things with me and shares problems with me; I am satisfied that my family accepts and supports my wishes to take on new activities or directions; I am satisfied with the way my family expresses affection and responds to my emotions; I am satisfied with the way my family and I share time together [19]. Each item has three response choices: “Often such” comments two points, “Sometimes this”comments one point, “rarely” comments 0 point. The five dimension scores were calculated, higher scores indicate better family function. Total score of 0–6 points represented a obstacle to family function and 7 to 10 meant good family function, so the AS sample can be divided into two compare groups in this current study.

### Generic instrument

The GWBS is a generic instrument providing information about six aspects of SWB and it is widely used in health surveys in the general population [16]. This schedule contains 33 items–the first 14 items are 6 response option items rated on a 6-point scale, the next 4 items are 0–10 rating bars scored on a 10-point scale, and the last 15 items are criterion-type behavioral and self-evaluation items [20]. Internal consistency coefficient of reliability for GWBS is 0.912, which indicate that it is a homogeneous scale basically measuring a singular subscale or general subjective state [20]. In our study, we used the Chinese version of the General Well-Being Schedule putted forwarded by JH Duan [21]. After reversing the scoring on questions with high scores indicating negative attributes, the scores are summed for a total well-being score. Scoring for the first 18 items ranged from 14 to 116 with high scores signifying favorable responses [22]. This schedule assesses six hypothetical dimensions indicating H (Health, 2 items); E (Energy, 3 items); S (Satisfaction, 2 items); SH (Sad or Happy, 4 items); O (Control, 3 items); RT (Relaxation and Tension, 4 items).

### Disease-specific instruments

The Bath Ankylosing Spondylitis Functional Index (BASFI) and the Bath Ankylosing Spondylitis Disease Activity Index (BASDAI) were used in AS for clinical and research purposes. The BASFI consists of eight visual analogue scales dealing with physical function and two scales reflecting the patient’s ability to cope with daily activities [23]. The BASDAI consists of six visual analogue scales dealing with fatigue, spinal pain, joint pain, localized tenderness, and quality and quantity of morning stiffness (BASDAI and BASFI: 0 = best, 100 = worst score) [24]. BASFI ≥ 5 means good function, so the AS samples are divided into two subgroups according to BASFI ≥ 5 and BASFI < 5. Similarly, BASDAI ≥4 is defined as active stage. According to whether the value of BASDAI more than 4, the patients with AS are categorized into two groups. Visual Analog Scale (VAS), a numerical scale (11 points that initiate in 0 [no pain] and end in 10 [worst imaginable pain]), has been widely used in pain measurement of BASDAI [25]. This test has been certificated for a very long time and is widely accepted in many articles. It is reliable and easy to apply this scale in pain assessment.

### Statistical analyses

All statistical tests were carried out using SPSS version 16.0 for Windows. First, the socio demographic and clinical variables were subjected to descriptive analysis. The numerical variables were expressed as means and standard deviations (SD), and the categorical variables were expressed as absolute and relative frequencies. Differences between patients and the healthy controls were examined by *χ*^2^ tests of categorical variables. Independent-Samples *t*-test was used to compare the mean scores of the GWB scales in the patient group and the general population. Within the patient group, two-sample t tests or One-way ANOVA were used for comparisons. Multiple linear regression analyses were applied to study the predictors of SWB. The significant level was set at *p* < 0.05.

## Results

### Study samples

The characteristics of samples are illustrated in Table 1. The age of the healthy controls and the AS patients was comparable (*P* = 0.773), so was the sex ratio (*P* = 0.115). The proportion of physical workers was different, and the mean education level of the general population sample was higher than that of the AS sample (*P* < 0.001).

**Table 1:** Characteristics of patients with AS and the healthy controls

Characteristics	AS (*n* = 200)	HC (*n* = 210)	*P* value
Age (years), mean (SD)	29.54 (9.57)	29.24 (10.79)	0.773
Men (%)	165 (82.50)	160 (76.19)	0.115
Occupation			<0.001*
Mental workers (%)	93 (46.50)	167 (79.52)	
Physical workers (%)	107 (53.50)	43 (20.48)	
Educational level			<0.001*
≤ 12 years (%)	137 (68.50)	68 (32.38)	
> 12 Years (%)	63 (31.50)	142 (67.62)	
Place of residence (%)			0.232
Rural residence	120 (60.00)	113 (53.80)	
City residence (%)	80 (40.00)	97 (46.20)	
Family income			0.005*
> 1500 (%)	133 (66.50)	167 (79.52)	
≤ 1500 (%)	66 (33.00)	43 (20.48)	
Sleep quality			<0.001*
Better (%)	60 (30.00)	89 (42.38)	
General (%)	93 (46.50)	100 (47.62)	
Poor (%)	47 (23.50)	21 (10.00)	
Life Satisfaction			0.051
Particularly Satisfied (%)	13 (6.50)	26 (12.38)	
Satisfied (%)	145 (72.50)	153 (72.86)	
Not Satisfied (%)	42 (21.00)	31 (14.76)	
Therapy Prospect (attitudes toward therapy)			
Very Optimistic (%)	14 (7.0)		
Optimistic (%)	84 (42.0)		
General (%)	84 (42.0)		
Poor (%)	13 (6.5)		
No Hope (%)	5 (2.5)		
Disease duration			
≤ 5 years (%)	134 (67.00)		
> 5 years (%)	66 (33.00)		
APGAR, mean (SD)	6.95 (2.25)		
BASDAI, mean (SD)	2.71 (2.03)		
BASFI, mean (SD)	1.68 (1.94)		

*AS* ankylosing spondylitis, *HC* healthy controls, *APGAR* Adaptation, Partner-ship, Growth, Affection and Resolve, *BASDAI* Bath Ankylosing Spondylitis Disease Activity Index, *BASFI* Bath Ankylosing Spondylitis Functional Index, *mean* average value, *SD* standard deviation

*P < 0.05

**Fig. 1:**
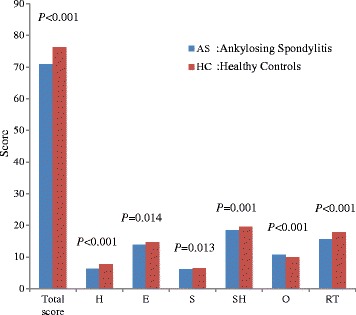
The comparison for each of the subscales between AS patients and healthy controls

The patients reported significantly worse health on all scales of the GWB compared with the general population except for the Control (O) scale (Table 2 and Fig. 1).

**Table 2:** Summary of GWB domains

Subscale	AS, mean (SD)	HC, mean (SD)	*P* value
H	6.25(2.04)	7.67(2.37)	<0.001
E	13.81(3.23)	14.60(3.21)	0.014
S	6.17(1.40)	6.50(1.23)	0.013
SH	18.41(3.91)	19.63(3.39)	0.001
O	10.78(1.99)	10.02(1.95)	<0.001
RT	15.57(3.25)	17.90(3.47)	<0.001
Total Score	70.98(11.12)	76.30(9.48)	<0.001

*AS* Ankylosing Spondylitis, *HC* healthy controls, *H* Health, *E* Energy, *S* satisfaction, *SH* Sad or Happy, *O* control, *RT* Relaxation and Tension, *mean* average value, *SD* standard deviation

The results of univariate analysis in the AS sample are shown in Table 3. Significant differences were found between physical workers and mental workers in the AS group for H scale (*p* = 0.015), SH scale (*p* = 0.034), with physical workers reporting worse health than mental workers (Table 3). Furthermore, there were significant correlations between the following variables and GWB scores, APGAR, life satisfaction, BASDAI, sleep quality, therapy prospects and place of residence with E (All *P* < 0.05); place of residence, life satisfaction, therapy prospects, APGAR, and educational level with S (All *P* < 0.05); All variables except educational level have been found to be correlated with SH (All *P* < 0.05). Therapy prospects and BASDI were associated with O (*P* = 0.037, *P* < 0.001). There are statistical correlations between RT and sleep quality, life satisfaction, therapy prospects, APGAR, BASDAI (*P* < 0.05). Furthermore, total score was positively related to APGAR (*P* < 0.001). It was negatively related to BASDAI (*P* < 0.001) and BASFI (*P* = 0.044). Sleep quality was also significant (*P* < 0.001), with better sleep showing higher SWB than worse sleep. Life satisfaction and therapy prospects were negatively related to SWB (All *P* < 0.001).

**Table 3:** The results of univariate analysis in AS group (shown as *P* value)

Predictors	H	E	S	SH	O	RT	Total score
Occupation	0.015*	0.067	0.113	0.034*	0.166	0.533	0.142
Place of residence	0.593	0.048*	0.041*	0.028*	0.203	0.395	0.047*
Educational level	0.742	0.425	0.014*	0.135	0.769	0.776	0.330
Life satisfaction	0.103	<0.001*	<0.001*	<0.001*	0.504	0.001*	<0.001*
Therapy Prospect	0.231	0.002*	0.020*	0.020*	0.037*	<0.001*	<0.001*
Sleep quality	0.274	<0.001*	0.192	0.001*	0.320	0.046*	<0.001*
Duration disease	0.599	0.131	0.528	0.016*	0.186	0.788	0.191
APGAR	0.181	<0.001*	0.011*	<0.001*	0.777	<0.001*	<0.001*
BASDAI	0.153	<0.001*	0.060	<0.001*	<0.001*	<0.001*	<0.001*
BASFI	0.896	0.306	0.480	0.006*	0.162	0.194	0.044*

*H* Health, *E* Energy, *S* satisfaction, *SH* Sad or Happy, *O* control, *RT* Relaxation and Tension

*P < 0.05

### Multiple linear regression analyses

Multivariable linear regression analysis showed that the following variables were significantly associated with the score of six subscales: Occupation, BASDAI, Life satisfaction and Sleep quality with H (All *P* < 0.05); Sleep quality,therapy prospects APGAR and BASDAI with E (All *P* < 0.05); life satisfaction (*P* = 0.002), therapy prospects (*P* = 0.039), APGAR (*P* = 0.019) with S; Life satisfaction (*P* = 0.038), therapy prospects (*P* < 0.001), Sleep quality (*P*=0.013) APGAR (*P* < 0.001), BASDAI (*P* = 0.010) with SH; BASDAI (*P* = 0.013) with O; therapy prospects (*P* = 0.002), APGAR (*P* = 0.001), BASDAI (*P* = 0.001) with RT.

However, final multivariable linear regression analysis with total score as dependent variable included four predictors (see Table 4). The significant model obtained, revealed that better sleep, lower disease activity and more family care predicted higher SWB. Therapy prospect was also a significant predictor in this model with positive attitudes reporting higher well-being than negative attitudes.

**Table 4:** Multivariable linear analysis of association between demographics, APGAR and BASDAI with the GWB

Predictors	H		E		S		SH		O		RT		Total	Score
	*β*	*P*	*β*	*P*	*β*	*P*	*β*	*P*	*β*	*P*	*β*	*P*	*β*	*P*
Occupation	0.822	0.005*	−0.464	0.643	−0.057	0.398	−0.033	0.596	−0.076	0.288	0.040	0.544	0.015	0.805
Life satisfaction	0.879	0.003*	−1.130	0.260	−0.618	0.002*	−1.033	0.038*	−0.049	0.501	−0.125	0.070	−0.088	0.182
Therapy Prospect	−0.018	0.808	−0.775	0.002*	−0.250	0.039*	−1.288	<0.001*	−0.113	0.118	−0.808	0.002*	−3.410	<0.001*
Sleep quality	−0.435	0.032*	−1.052	<0.001*	−0.042	0.546	−0.827	0.013*	−0.038	0.595	−0.091	0.174	−2.915	0.002*
APGAR	0.084	0.231	0.315	0.001*	0.103	0.019*	0.395	<0.001*	−0.023	0.746	0.324	0.001*	1.158	<0.001*
BASDAI	−0.158	0.032*	−0.211	0.047*	−0.086	0.222	−0.313	0.010*	−0.176	0.013*	−0.352	0.001*	−1.192	0.001*

*APGAR* Adaptation, Partner-ship, Growth, Affection and Resolve, *BASDAI* Bath Ankylosing Spondylitis Disease Activity Index, *H* Health, *E* Energy, *S* satisfaction, *SH* Sad or Happy, *O* control, *RT* Relaxation and Tension

*P < 0.05

## Discussion

In this study, we aimed to determine which variables affect the SWB assessed by GWBS survey in AS patients. The significant correlation in the AS group was obtained between the four variables (sleep quality, Family-APGAR, BASDAI, therapy prospect) and SWB.

Firstly, in this Subjective Well-being survey, patients with AS reported significantly impaired health on all scales of GWB except for the O scale, compared with the healthy controls. Age and sex ratio are comparable in the two samples, thus, we consider that SWB may be affected by the AS disease.

Secondly, the results of single factor analysis in the AS patients indicated that SWB may differ significantly between two subgroups of residence. In addition, the SWB of physical workers differed significantly from the SWB of mental workers. However, when analyzing demographic variables in the healthy population, age, marital status, family income and occupation have been found to be associated with the total score of SWB (*P* = 0.004, *P* = 0.001, *P* = 0.003, *P* = 0.013). That is to say, demographic variables do not appear to have much of an impact on SWB in the AS patients, which is consistent with the previous study in healthy people [26]. Study on patients attending community-based mental health services reported that demographic variables explained only 2.9 % of the variance in subjective quality of life [27].

Thirdly, the regression analyses revealed that subjective well-being was positively associated with better sleep, lower disease activity and more family care, therapy prospect. Occupation and place of residence were excluded from the regression equation, which highlighted the fact that demographic variables did not play a crucial role in the well-being of AS patients.

We observed that 23.5 % of participants suffered from sleep disorder, and sleep quality, which should not be ignored, has been found to affect SWB in AS patients. In addition, some results have also been reported that there was a higher rate of sleep disturbance in patients with AS [28, 29], which were important concerns in patients with AS [30]. In agreement with these findings, recent studies [31, 32] have suggested that poor sleep can impair well- being, most of these studies have involved individuals’ perceptions of sleep quality and duration. However, Jean-Louis and colleagues [33] found no association between sleep quality and SWB in adult general population. Sleep disturbance is often produced by inflammatory pain [5, 34]. Also, mental as well as physical aspects were affected due to the poor sleep quality [35]. Consequently, it is not hard to follow the relationship between sleep quality and SWB in this study. We all know that, poor sleep quality can aggravate the patients’ condition, which is unfavorable for the recovery of the disease. Sleep disturbance is frequently complained by patients with AS and is still largely ignored by clinical care and research [36]. Thus, we should pay great attention to the patients’ quality of sleep, to examine the independent risk factors of sleep quality and to improve sleep quality in AS patients.

Family function is what family performs on the behalf of its members in a larger society and it is assessed by APGAR [37]. In our study, family function appeared to be related to SWB in AS patients. Previous study suggested that family function had much effect on quality of life and well-being [37]. Likewise, Andrea reported that family ties had significant and positive associations with psychological well-being [38]. Kenneth et al. [39] also confirmed that family function in women with rheumatoid arthritis is related to subjective well-being, beyond the pain and fatigue associated with SWB. To our knowledge, Family care or Family-APGAR to some extent can help us cope with stress, anxiety and various emergencies, which could influence SWB. It should be considered as a determinant of health to improve subjective well-being. In addition, BASDAI has a negative relationship with the variable of subjective well‑being. BASDAI addresses disease activity which definitely affects quality of life, thereby, it is reasonable to draw such a conclusion. The conclusion is highly consistent with Bing Han’s research indicating functional capacity as predictor of psychological health [40], as well as the study of Geertzen which concluded that the influence on SWB was less when patients were physically independent [41]. Based on the findings of the present study, positive attitudes towards therapy prospect leads to higher well‑being of the patients with AS. To our knowledge, it is necessary to stay positive and then to be helpful to our state of mind.

Lastly, there are some limitations of our study. First, we recruited AS patients from one hospital, even though the hospital has a wide audience in the province, findings of this study cannot be generalized to all AS patients in our society. Second, it was a cross-sectional design and therefore can only be used to draw conclusions based on the relationships among variables. Longitudinal studies should be performed to identify the effects found in the present study. Third, although we have found sleep quality had significant correlations with SWB, many influencing factors of sleep quality should be explored. Last, we did not consider the relationship between SWB and kinds of drugs patients accepted, which should be verified in further studies.

## Conclusions

SWB was significantly associated with sleep quality, therapy prospects, APGAR and BASDAI. It is important for clinicians to be aware of complicated relationships between clinical variables and SWB. In order to perfect AS patients’ SWB, current management strategies should focus on reducing disease activity, improving sleep quality and family ties.

## Abbreviations

AS: Ankylosing spondylitis
VAS: Visual analogue scale
BASDAI: Bath Ankylosing Spondylitis Disease Activity Index
BASFI: Bath Ankylosing Spondylitis Functional Index
APGAR: Adaptation, Partner-ship, Growth, Affection and Resolve
GWB: General well-being
SWB: Subjective well-being
HRQOL: Health-related quality of life
